# Supplementary material for: Impact of Type II LRRK2 inhibitors on signaling and mitophagy
Source: Biochem J. 2021 Oct 6;478(19):3555–73. doi: 10.1042/BCJ20210375 (PMC8589421; doi:10.1042/BCJ20210375)

Supplementary Figure 1A

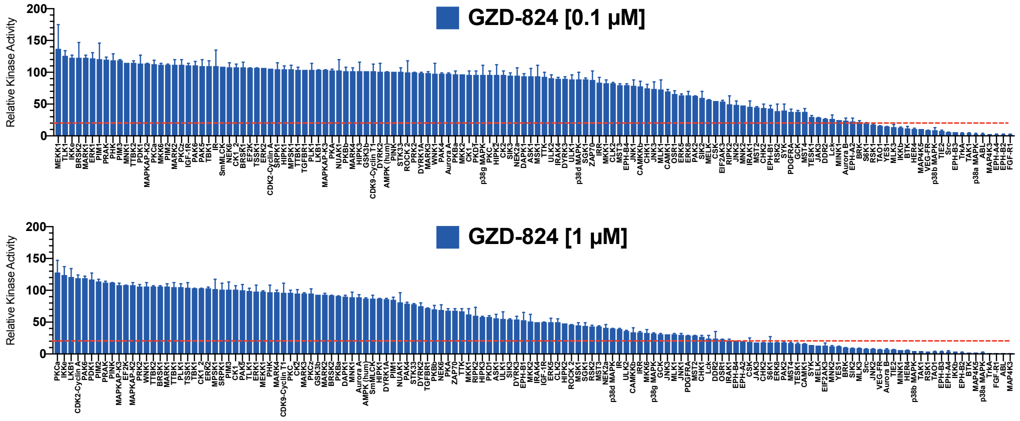

Supplementary Figure 1B

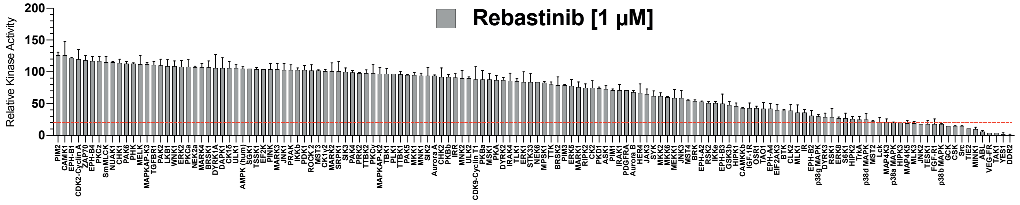

Supplementary Figure 1C

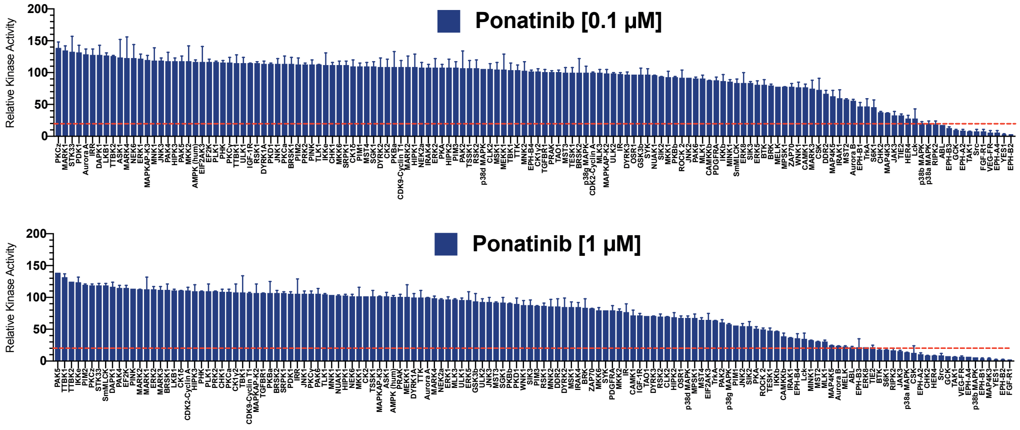

Supplementary Figure 1D

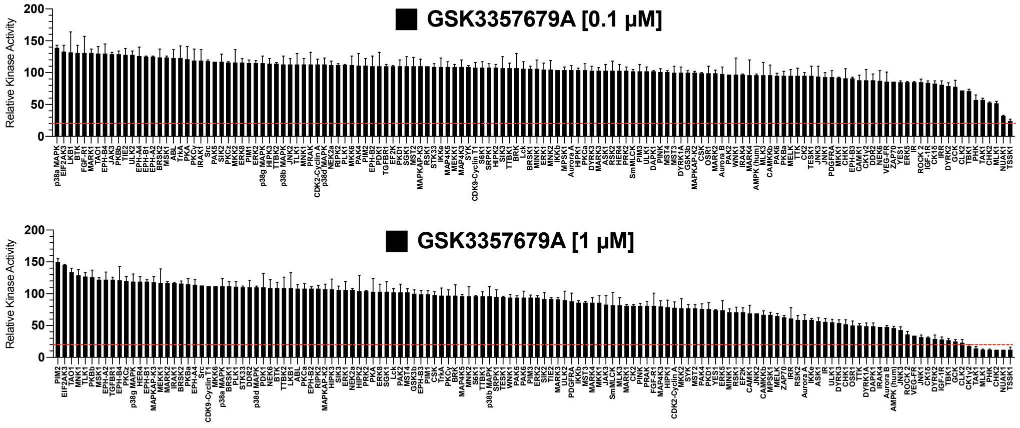

Supplementaryrary Figure 2

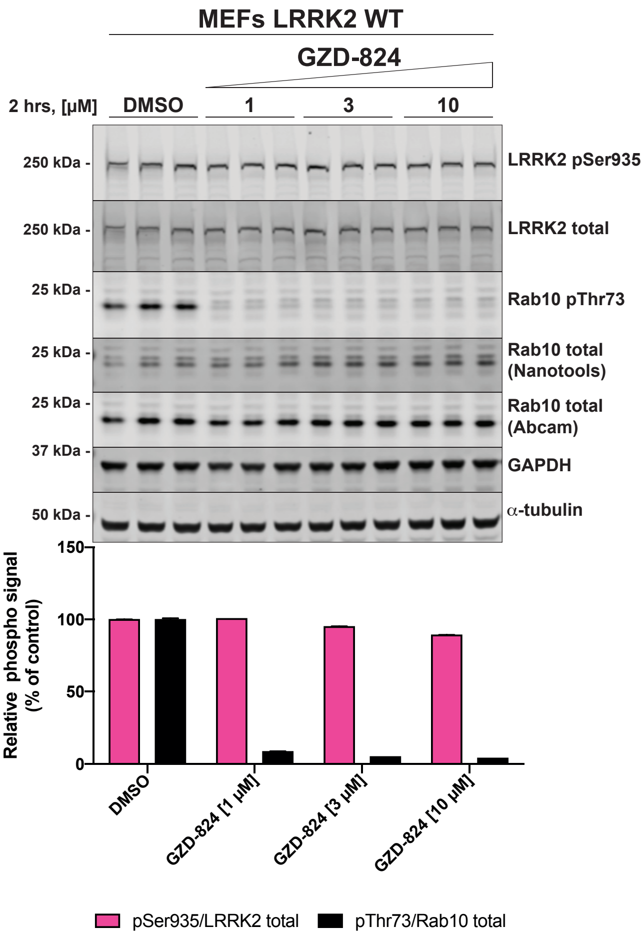

Supplementary Figure 3

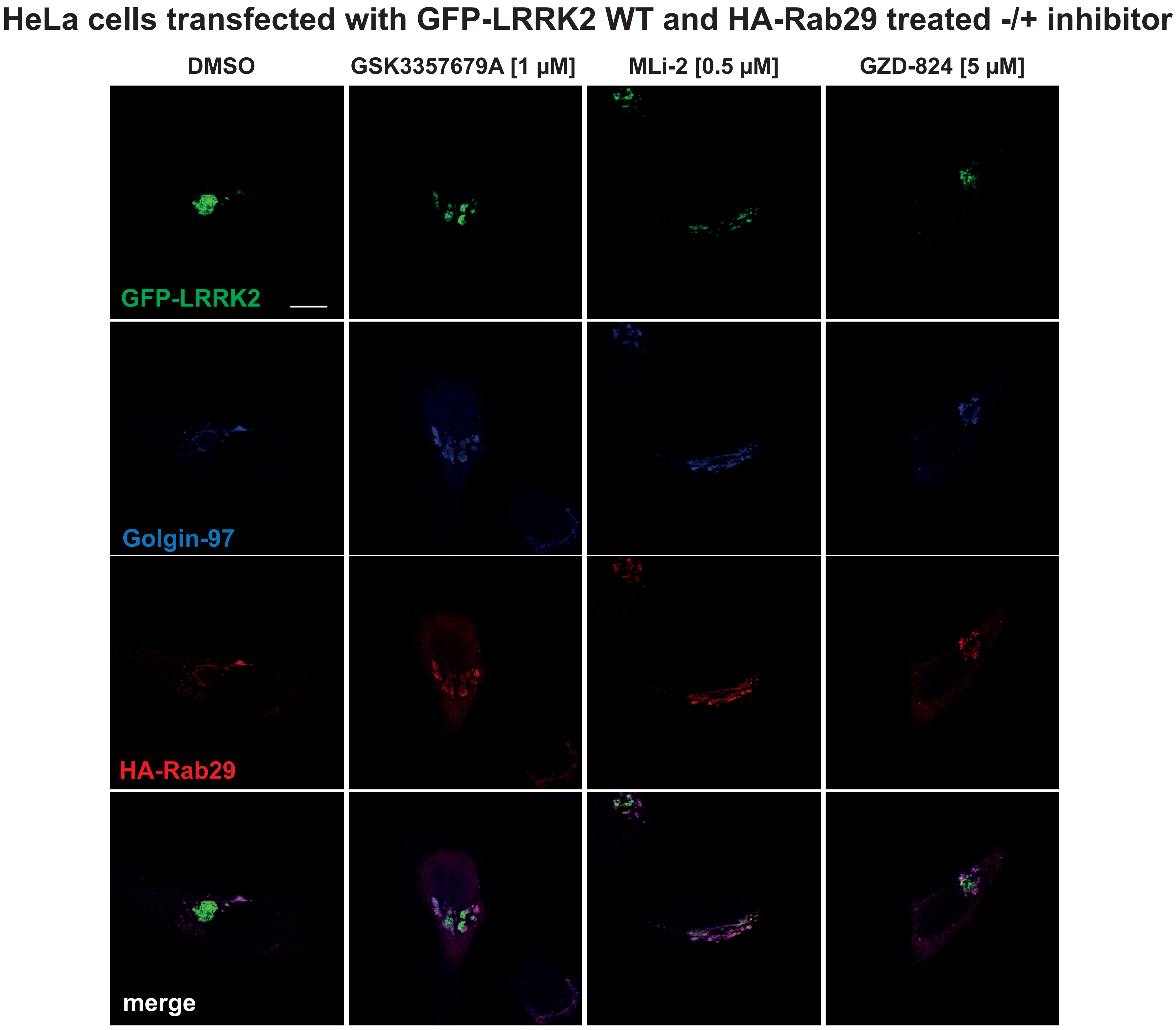

Supplementary Figure 4

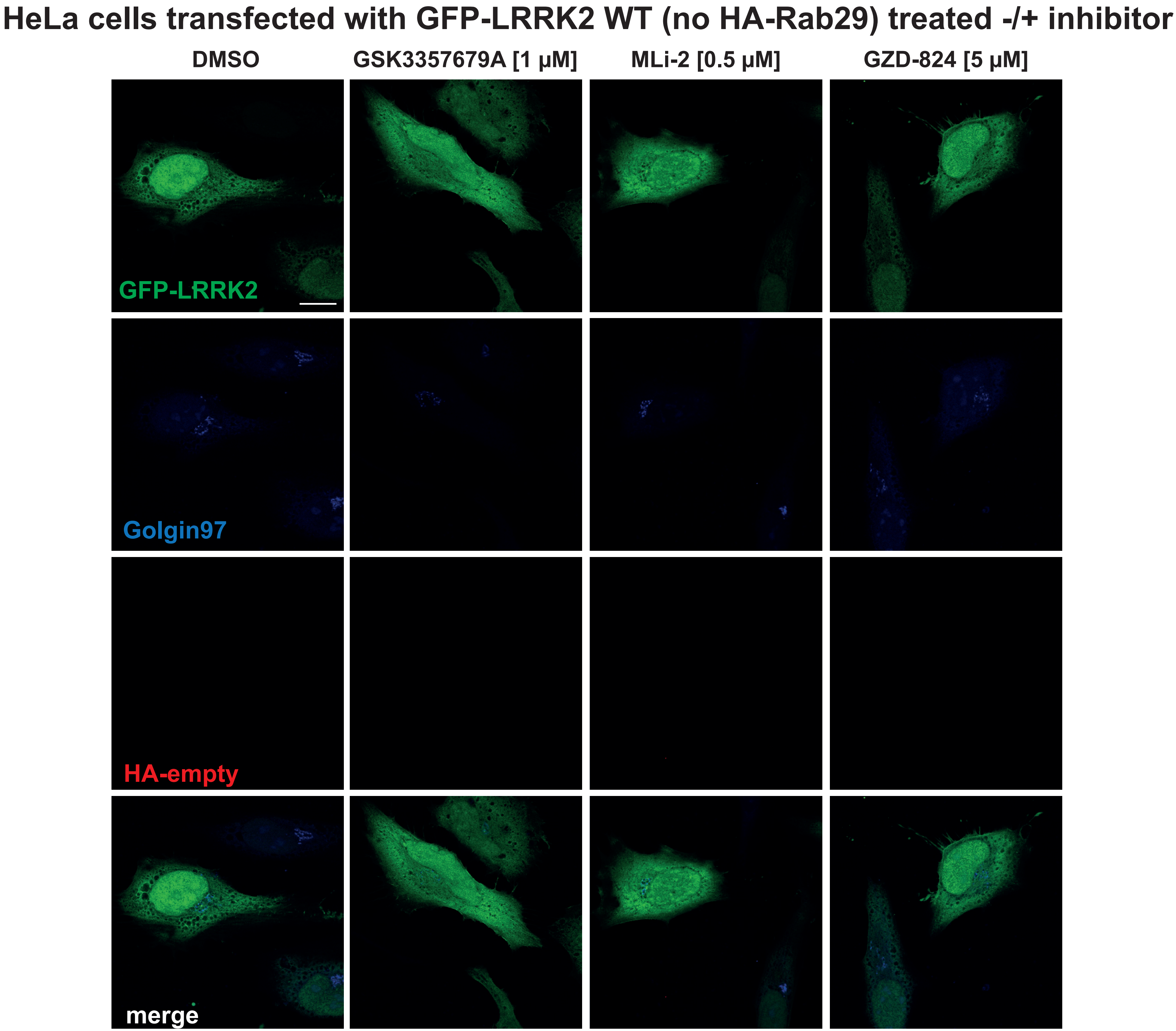

Supplement: Supplementary Figures S1-S4 [file BCJ-478-3555-s1.pdf]
